# From Resolution to Revolution: Cryo-EM Driven Advances in Structure-Based Drug Design for Membrane Proteins and Targeted Protein Degradation

**DOI:** 10.1063/4.0001146

**Published:** 2025-10-27

**Authors:** Victoria Ouroutzoglou

**Affiliations:** Viva Biotech, Cambridge, MA, USA

## Abstract

Cryo-electron microscopy (cryo-EM) has transformed Structure Based Drug Discovery by enabling high-resolution visualization of previously intractable protein structures in their native, dynamic states. This technology has provided insights into drug targets that were previously too challenging to study, thus accelerating the drug discovery process. With the ‘Resolution Revolution’ researchers were able to visualize atomic- level details of liganded protein structures of complex and large membrane proteins like G protein-coupled receptors (GPCRs), ion channels and transporters which are key drug targets. Other than traditional small molecule hit finding strategies other therapeutic modalities became more amenable for Structure Based Drug Discovery including oligo and peptide based therapeutics, biologics and protein/antibody drug conjugations (ADC). A promising and emerging area of active research focuses on Targeted Protein Degradation (TPD) and Glue like molecules, where understanding the Structure Activity Relationship (SAR) of these ternary models are becoming more critical to achieving effective target degradation. The following poster highlights how Protein Scientists and Structural Biologists at Viva Biotech are using these techniques to support drug development programs in our Membrane Protein and TPD/Glue platforms.

